# Coupling the fermentation and membrane separation process for polyamides monomer cadaverine production from feedstock lysine

**DOI:** 10.1002/elsc.202000099

**Published:** 2021-06-10

**Authors:** Ruoshi Luo, Zhao Qin, Dan Zhou, Dan Wang, Ge Hu, Zhiguo Su, Suojiang Zhang

**Affiliations:** ^1^ State Key Laboratory of Coal Mine Disaster Dynamics and Control Chongqing University Chongqing P. R. China; ^2^ Department of Chemical Engineering School of Chemistry and Chemical Engineering Chongqing University Chongqing P. R. China; ^3^ Institute of Process Engineering Chinese Academy of Sciences Beijing P. R. China

**Keywords:** batch fermentation, cadaverine, lysine decarboxylases, membrane separation, polyamides monomer

## Abstract

Nylon is a polyamide material with excellent performance used widely in the aviation and automobile industries, and other fields. Nylon monomers such as hexamethylene diamine and other monomers are in huge demand. Therefore, in order to expand the methods of nylon production, we tried to develop alternative bio‐manufacturing processes which would make a positive contribution to the nylon industry. In this study, the engineered *E. coli*‐overexpressing Lysine decarboxylases (LDCs) were used for the bioconversion of l‐lysine to cadaverine. An integrated fermentation and microfiltration (MF) process for high‐level cadaverine production by *E. coli* was established. Concentration was increased from 87 to 263.6 g/L cadaverine after six batch coupling with a productivity of 3.65 g/L‐h. The cadaverine concentration was also increased significantly from 0.43 g cadaverine/g l‐lysine to 0.88 g cadaverine/g l‐lysine by repeated batch fermentation. These experimental results indicate that coupling the fermentation and membrane separation process could benefit the continuous production of cadaverine at high levels.

**Abbreviations**
*C. glutamicum*
: *Corynebacterium glutamicum*
DAP: 1,5‐diaminopentane
*E. coli*
: *Escherichia coli*
LDCs: lysine decarboxylases
MF: microfiltration
OD_600_
: optical density at 600 nm
PAs: polyamides

## 1 INTRODUCTION

Nylon polyamides (PAs) are well‐known lightweight and tough synthetic polymers, which have been applied in a wide range of industries, including automotive, engineering plastics, electronics and electrical, textile, and others [1]. PA66 is one of the most important and widely used nylon varieties as it has strong dielectric resistance in thermally‐ and mechanically‐stressed moldings. It can be produced through chemical synthesis from fossil fuel resources, which cause environmental pollution [2]. However, biosynthesis of nylon monomer, which uses renewable raw materials as a green and safe production mode, can reduce environmental pollution and greenhouse gas emissions. Due to steadily growing demand, people are trying to find ways to produce nylon monomers by biological methods, such as cadaverine [3] and diprotic acid [4] to form biobased nylon PA56, which sheds light on the development of the renewable nylon market. Cadaverine, 1,5‐diaminopentane (DAP), a widely active nitrogen‐containing base in organisms, promises broad prospects for various applications, especially as an important monomer for bio‐based polyamides, owing to its characteristics. The cadaverine‐based polyamide, PA5X [5], suggests an extremely competitive market potential, due to the high consumption of engineered plastics and fibers. For example, nylon PA56 has remarkable properties such as its light weight, good moisture absorption, thermo‐resistance, high tensile strength, low temperature dyeing, high elasticity, and flame retardancy [6].

**FIGURE 1:**
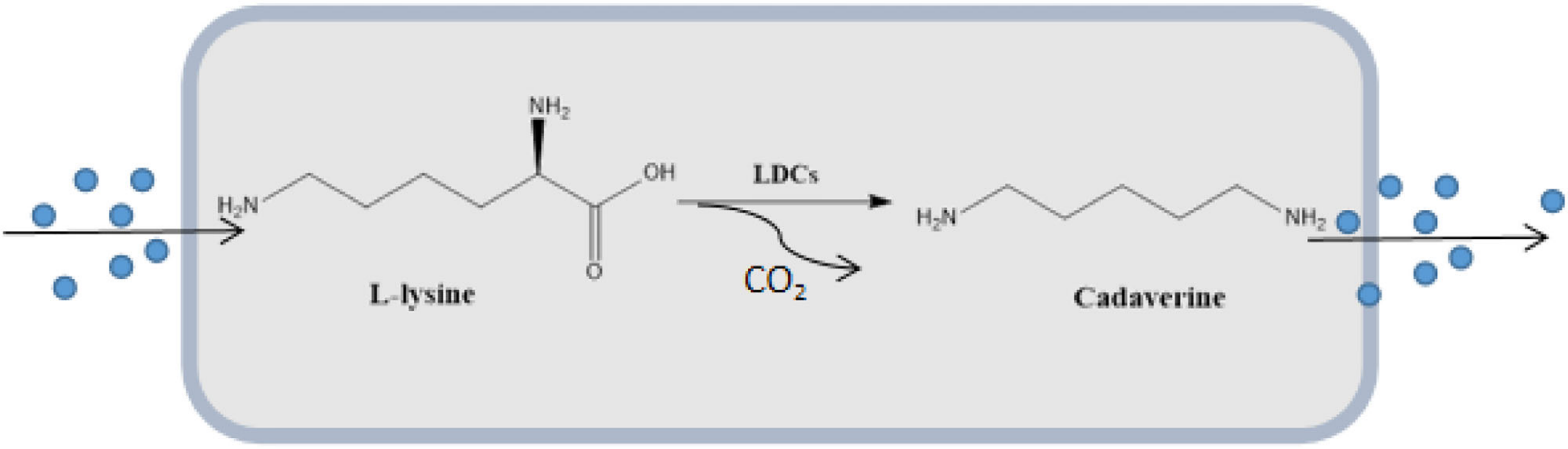
The production of DAP. l‐lysine was converted to 5t6r by the action of lysine decarboxylase

DAP can be biologically produced by a one‐step enzymatic conversion of l‐lysine enabling the intracellular decarboxylation of the l‐lysine to DAP (Figure 1). There are two well‐known examined lysine decarboxylases (LDCs) in *Escherichia coli*, encoded by *cadA* and *ldcC* [7]. Between them, the LDC encoded by *cadA* is more desirable than *ldcC* because it has twice the biological activity of *ldcC* [8]. *CadA* has been widely used in whole‐cell biotransformation and direct microbial fermentation [9]. Initial studies have proved that strains are mainly engineered from the conventional l‐lysine producers *Corynebacterium glutamicum* (*C. glutamicum*) [10] and *Escherichia coli* (*E. coli*) [3], which can produce DAP from renewable carbon resources and achieve potentially high level yields [6, 11]. Therefore, the biosynthesis of pentanediamine is promising.

However, several constraints need to be tackled for fermentative microbial production, such as the tolerance of the microbial system with respect to products which showed that the decline of productivity could result from feedback inhibition. In the fermentation process of pentane diamine, the product has a significant inhibitory effect on the growth and metabolism of the bacteria, resulting in a decrease in the product yield [12]. And the LDC encoded by *cadA* is a protein that becomes inactive when pH increases due to the high production of cadaverine [13, 14]. Therefore, finding a way to release the limits in tolerance of the product would be useful for producing cadaverine.

The fermentation‐separation coupling technology is the in‐situ coupling of the fermentation and separation processes to achieve enhancement, which can remove the product during producing [15, 16, 17]. This strategy has also been used to improve the productivity of lactic acid [18], succinic acid [19] and butanol [20]. The membrane can achieve separation under mild conditions at room temperature and low pressure (<0.2 MPa). That is, the semi‐permeability of the membrane is used to separate the components in the biological reaction system within a certain range of molecular size. A membrane device is used in the separation of the biological reaction process to separate cells, enzymes and some reactants from the reaction system [21, 22]. By selecting a semi‐permeable membrane with a suitable pore size, the product can be transmitted selectively.

**PRACTICAL APPLCATION**

Cadaverine,1,5‐diaminopentane (DAP), a widely active nitrogen‐containing base in organisms, exhibits broad prospects for various applications, especially as an important monomer for bio‐based polyamides owing to its environmentally friendly characteristics. Cadaverine is the most important precursor for nylon PA5X, which has an extremely competitive market due to the high consumption of engineered plastics and fibers. Therefore, the biosynthesis of pentanediamine is promising.

In the production of DAP, cells and macromolecules were intercepted by the membrane module and separated from reactants and cadaverine. Then cadaverine was adsorbed by the ultra‐highly cross‐linked resin, while the majority of the fermentation broth, which included the unconverted glucose and other nutrients, was recycled back into the fermenter. In this way, both reducing the inhibition of products on microorganisms and improving the efficiency were achieved to produce DAP [23].

In this study, the engineered *E. coli*‐overexpressing CadA‐B, CadAB‐B and CadAA‐B [24] were used for the bioconversion of l‐lysine to cadaverine, as shown in materials and methods. We established an integrated fermentation and microfiltration process for high‐level cadaverine production. The process circumvented the problem of production inhibition, enabled recycling of cells, and improved cadaverine production. Aiming to provide insights into the influence of different parameters on biotransformation kinetics, key factors of bioconversion such as substrate concentration, temperature and initial pH of fermentation were optimized for efficient reaction. Thus, the recycling of the biocatalyst can be performed by coupling the fermentation and membrane separation process, thereby improving the economy of the bioconversion system for largescale industrial application purposes.

## 2 MATERIALS AND METHODS

### 2.1 Plasmids and media

The *E. coli* BL21(DE3) was used as the origin strain in this study. The plasmid carrying a wild type of inducible lysine decarboxylase encoded by cadA was constructed as CadA‐B. The plasmid carrying CadA‐ldcC‐KivD(α‐Ketoacid decarboxylase) fusion protein was constructed as CadAB‐B and CadAA‐B encoded a high‐efficiency LDC screened by our laboratory saturation mutations. All the plasmids were codon optimized, chemically synthesized by Genewiz Co. (Suzhou, China), inserted into the BamHI/EcoRI restriction sites and cloned into the pACYC‐ Duet1 vector (Invitrogen, Waltham, MA, USA). Then, the plasmids were introduced into BL21(DE3) to form the novel recombinant CadA‐B, CadAB‐B and CadAB‐B. All the sequences of the plasmids constructed in this study are shown in supporting information.

The LB medium and M9 medium were used for strain growth and fermentation, respectively. An inoculation volume of 5% was used. Glucose was sterilized separately at 115°C for 30 min. The chemicals used in this study were of analytical grade and were from OXOID (England) or from Sinopharm Chemical Reagent Beijing Co., Ltd. (China), unless otherwise indicated.

### 2.2 Bioconversion procedures

The fermentation media were developed for evaluating the strain's potential for cadaverine production. The medium was supplemented by 10 g/L NaCl, 10 g/L tryptone, and 5 g/L yeast extract. For cadaverine production, a single colony of the desired strain was cultivated for 12 h at 37°C and 250 RPM in 2 mL LB medium supplemented with appropriate antibiotics. This starter culture was then transferred into 50 mL of fermentation medium supplemented with appropriate antibiotics, 1.0 mM MgSO_4_ and 0.5 mM ThDP at 37°C with 250 RPM orbital shaking at a starting optical density at 600 nm (OD_600_) of 0.1 in a 250 mL flask. After an OD_600_ of 0.6 had been reached, 0.5 mM of IPTG and 20 g/L of l‐lysine were added. Flasks were then incubated at 30°C.

### 2.3 Protein expression

BL21(DE3) harboring *E. coli* CadA‐B, CadAB‐B and CadAA‐B were screened on selective LB agar plates supplemented with 100 μg/mL ampicillin, individually. Positive clones were inoculated in 2 mL LB at 37°C and 250 RPM for 12 h. One milliliter of seed cultures were transferred into 50 mL M9 containing 100 μg/mL ampicillin. At an OD_600_ of 0.6, the cultures were induced at a final concentration of 0.5 mM IPTG, and incubated for 16 h at 30°C.

### 2.4 Membrane repeated batch fermentation (MRBF)

A hollow‐fiber MF membrane model (nominal pore size of 0.45 μm, total area of 0.09 m^2^) was used in this study (Damotech Co., Ltd. Kr.). After each run, the membrane was cleaned, first with 1 mol/L NaOH, water, 1 mol/L HCl solutions and then water at room temperature. MF equipment and columns were sterilized with 0.2 g/L sodium hypochlorite (NaClO) solution. Seed tanks, fermentation tanks, buffer tanks, storage tanks, and connecting pipes were sterilized by autoclaving at 121°C prior to fermentation. The filtrate passed through an adsorption column equipped with ultra‐high cross‐linked resin, is then adsorbed and washed to remove impurities, and is desorbed with adipic acid aqueous solution as a desorbent. The ultra‐highly cross‐linked resin with good hydrophilicity is based on polystyrene‐divinylbenzene. After washing with deionized water, the resin is ready for reuse. The columns are also sterilized with 0.2 g/L NaClO solution, and then rinsed with deionized water.

### 2.5 Analytical methods

The quantification of cadaverine was conducted by high performance liquid chromatography (HPLC) using a 1260 system (Agilent Co., Ltd, CA, USA) with an Agilent Eclipse XDB‐C18 column (4.6 mm × 150 mm × 5 μm). For liquid chromatography‐mass spectrometry (LC‐MS) identification of cadaverine, exact mass spectra were explored with a Bruker micrOTOF‐Q II mass spectrometer using the time of flight (TOF) technique, equipped with an ESI source operating in negative mode (Burker Co., Ltd, USA). The product was verified by LC–MS.

## 3 RESULTS AND DISCUSSION

### 3.1 Heterologous expression of the predicted lysine decarboxylases

**FIGURE 2:**
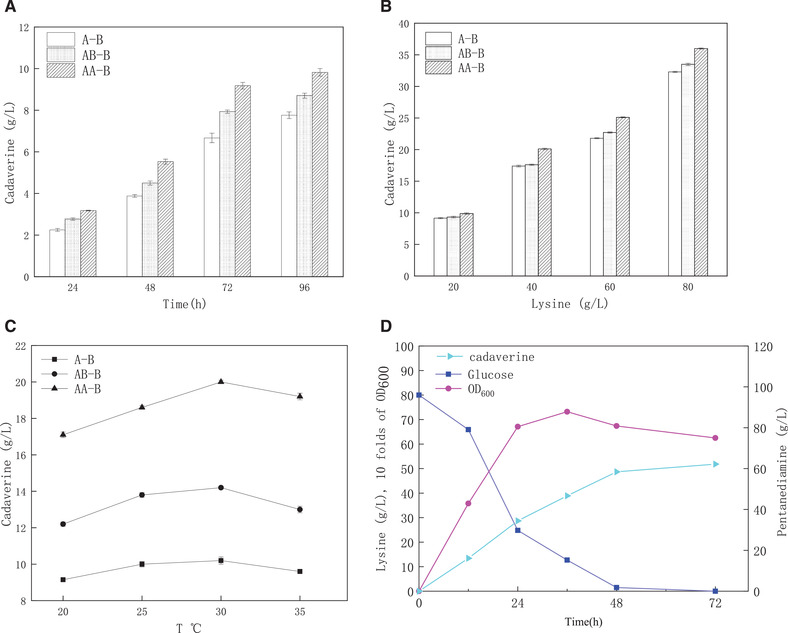
Key factors bioconversion such as substrate concentration, temperature and time of fermentation were optimized for efficient reaction. (A) The final quantity of cadaverine produced by three lysine decarboxylases fermentation (pH = 7, temperature was 30℃ and the concentration of cadaverine was 20 g/L). (B) The fermentations of 20, 40, 60, and 80 g/L l‐lysine. (pH = 7, temperature was 30℃ and fermentation time was 96 h) (C) The temperature effecting cadaverine. (pH = 7, the concentration of cadaverine was 40 g/L) (D) The concentration of cadaverine and glucose in the broth with the change of OD_600_ over the course of 72 h. (pH = 7, temperature was 30℃ and the concentration of cadaverine was 90 g/L)

l‐Lysine was converted to cadaverine by the action of lysine decarboxylase. (Figure 1) As shown in Figure 2A, the final quantity of cadaverine produced by three lysine decarboxylases was 7.76, 8.7, and 9.8 g/L with 20 g/L l‐lysine. These results indicated that the highly saturated mutant lysine decarboxylases expressed in the *E. coli* strain produced large amounts of cadaverine under appropriate conditions. In the present work, all the fermentations were successfully conducted under sterilized conditions. The result showed that the engineered CadAA‐B could express lysine decarboxylase that convert l‐lysine to cadaverine more efficiently (Figure 2A), which proves that the lysine decarboxylase obtained by the experimental screening is more efficient. During the fermentation of 20, 40, 60, and 80 g/L l‐lysine, the obtained cadaverine concentrations were 9.9, 20.1, 25.2, and 36.1 g/L, respectively. (Figure 2B) Figure 2C shows that 30°C is the optimal temperature for cadaverine synthesis. And Figure 2D reveals that the OD_600_ has changed by the time of fermentation while the concentration of glucose in broth decreased to a low level after 36 h of fermentation. Furthermore, it was shown that the cadaverine fermentation in repeated fed‐batch cultivation and l‐lysine level was supplemented to 120 g/L over the course of 96 h. Meanwhile, a maximum cadaverine concentration was achieved as 87.0 g/L (Figure  2A). However, the production of cadaverine was not sufficient due to significant product inhibition.

### 3.2 Membrane separation and repeated fed‐batch cadaverine fermentation

**FIGURE 3:**
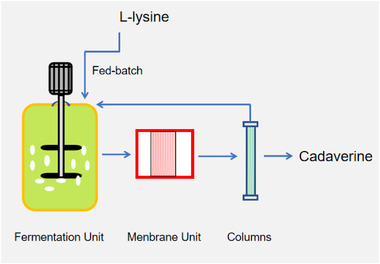
Device schematic of coupling the fermentation and membrane separation process

**FIGURE 4:**
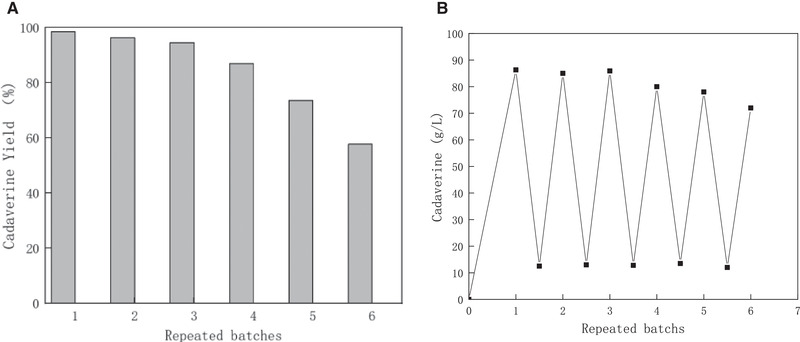
The progress of batch fermentation coupling the fermentation and membrane separation. The concentration in broth of cadaverine in six batch of membrane separation and fermentation. (pH = 7, temperature was 30℃ and time in each batch was 12 h)

The membrane was used during the fermentation (Figure 3). After membrane filtration, the OD value of the microfiltered broth was almost zero, indicating that the membrane completely retained the microbial cells. The increased cadaverine concentration in broth generally resulted in an increase of in permeate while the percent conversion was decreased in repeated batches. (Figure 4A) The cadaverine concentration in permeate was high enough for separation.

### 3.3 Integrated process for membrane fermentation and adsorptive separation

After integrated process for membrane fermentation and adsorptive separation, the concentrations of cadaverine in permeate were maintained in a range of 80–90.1 g/L with a productivity of 3.65 g/L‐h, respectively (Figure 4B). All the results proved the potential superiority of coupling the fermentation and membrane separation process.

## 4 CONCLUDING REMARKS

A novel coupling of the fermentation and membrane separation process was developed to improve the productivity of cadaverine with high‐yield lysine decarboxylases. In the process, the concentration of cadaverine generated from 87 g/L cadaverine to 263.6 g/L cadaverine significantly after six batches coupling with a productivity of 3.65 g/L‐h. The process circumvented the problem of production inhibition, enabled recycling of cells, and improved productivity of cadaverine production. Therefore, this advanced membrane coupled process can effectively facilitate bio‐nylon production. And this strategy of coupling the fermentation and membrane separation processes can be used in the production of other molecules, as well.

## CONFLICT OF INTEREST

The authors have declared no conflicts of interest.

## ETHICAL APPROVAL

This article does not contain any studies with human participants or animals performed by any of the authors.

## Supporting information

Supporting Information

## Data Availability

The data that support the findings of this study are available from the corresponding author upon reasonable request.
