# Supplementary material for: Coupling the fermentation and membrane separation process for polyamides monomer cadaverine production from feedstock lysine
Source: Eng Life Sci. 2021 Jun 10;21(10):623–9. doi: 10.1002/elsc.202000099 (PMC8518567; doi:10.1002/elsc.202000099)
Supplement: Supplementary file 1 — Supporting Information [file ELSC-21-623-s001.pdf]

Supporting information for the manuscript

"Coupling the Fermentation and Membrane Separation Process for Polyamides Monomer Cadaverine Production from Feedstock Lysine"

Ruoshi Luo[1,2], Zhao Qin[2], Dan Zhou[2], Dan Wang[1,2*], Ge Hu[2*], Zhiguo Su[3], Suojiang Zhang[3]

Supplementary Tables

Table S. Nucleotide sequences of codon optimized gene for over-expression used in this work.

>cadA lysine decarboxylase 1 [Escherichia coli str. K-12 substr. MG1655]

ATGAACGTTATTGCAATATTGAATCACATGGGGGTTTATTTTAAAGAAGAAC
CCATCCGTGAACTTCATCGCGCGCTTGAACGTCTGAACTTCCAGATTGTTTA
CCCGAACGACCGTGACGACTTATTAAAACTGATCGAAAACAATGCGCGTCT
GTGCGGCGTTATTTTTGACTGGGATAAATATAATCTCGAGCTGTGCGAAGAA
ATTAGCAAAATGAACGAGAACCTGCCGTTGTACGCGTTCGCTAATACGTATT
CCACTCTCGATGTAAGCCTGAATGACCTGCGTTTACAGATTAGCTTCTTTGA
ATATGCGCTGGGTGCTGCTGAAGATATTGCTAATAAGATCAAGCAGACCACT
GACGAATATATCAACACTATTCTGCCTCCGCTGACTAAAGCACTGTTTAAAT
ATGTTCGTGAAGGTAAATATACTTTCTGTACTCCTGGTCACATGGGCGGTAC
TGCATTCCAGAAAAGCCCGGTAGGTAGCCTGTTCTATGATTTCTTTGGTCCG

AATACCATGAAATCTGATATTTCCATTTCAGTATCTGAACTGGGTTCTCTGCT
GGATCACAGTGGTCCACACAAAGAAGCAGAACAGTATATCGCTCGCGTCTT
TAACGCAGACCGCAGCTACATGGTGACCAACGGTACTTCCACTGCGAACA
AAATTGTTGGTATGTACTCTGCTCCAGCAGGCAGCACCATTCTGATTGACCG
TAACTGCCACAAATCGCTGACCCACCTGATGATGATGAGCGATGTTACGCC
AATCTATTTCCGCCCGACCCGTAACGCTTACGGTATTCTTGGTGGTATCCCA
CAGAGTGAATTCCAGCACGCTACCATTGCTAAGCGCGTGAAAGAAACACC
AAACGCAACCTGGCCGGTACATGCTGTAATTACCAACTCTACCTATGATGGT
CTGCTGTACAACACCGACTTCATCAAGAAAACACTGGATGTGAAATCCATC
CACTTTGACTCCGCGTGGGTGCCTTACACCAACTTCTCACCGATTTACGAA
GGTAAATGCGGTATGAGCGGTGGCCGTGTAGAAGGGAAAGTGATTTACGA
AACCCAGTCCACTCACAAACTGCTGGCGGCGTTCTCTCAGGCTTCCATGAT
CCACGTTAAAGGTGACGTAAACGAAGAAACCTTTAACGAAGCCTACATGAT
GCACACCACCACTTCTCCGCACTACGGTATCGTGGCGTCCACTGAAACCGC
TGCGGCGATGATGAAAGGCAATGCAGGTAAGCGTCTGATCAACGGTTCTAT
TGAACGTGCGATCAAATTCCGTAAAGAGATCAAACGTCTGAGAACGGAAT
CTGATGGCTGGTTCTTTGATGTATGGCAGCCGGATCATATCGATACGACTGA
ATGCTGGCCGCTGCGTTCTGACAGCACCTGGCACGGCTTCAAAAACATCGA
TAACGAGCACATGTATCTTGACCCGATCAAAGTCACCCTGCTGACTCCGGG
GATGGAAAAAGACGGCACCATGAGCGACTTTGGTATTCCGGCCAGCATCGT
GGCGAAATACCTCGACGAACATGGCATCGTTGTTGAGAAAACCGGTCCGTA
TAACCTGCTGTTCCTGTTCAGCATCGGTATCGATAAGACCAAAGCACTGAG

CCTGCTGCGTGCTCTGACTGACTTTAAACGTGCGTTCGACCTGAACCTGCG
TGTGAAAAACATGCTGCCGTCTCTGTATCGTGAAGATCCTGAATTCTATGAA
AACATGCGTATTCAGGAACTGGCTCAGAATATCCACAAACTGATTGTTCAC
CACAATCTGCCGGATCTGATGTATCGCGCATTTGAAGTGCTGCCGACGATG
GTAATGACTCCGTATGCTGCATTCCAGAAAGAGCTGCACGGTATGACCGAA
GAAGTTTACCTCGACGAAATGGTAGGTCGTATTAACGCCAATATGATCCTTC
CGTACCCGCCGGGAGTTCCTCTGGTAATGCCGGGTGAAATGATCACCGAAG
AAAGCCGTCCGGTTCTGGAGTTCCTGCAGATGCTGTGTGAAATCGGCGCTC
ACTATCCGGGCTTTGAAACCGATATTCACGGTGCATACCGTCAGGCTGATGG
CCGCTATACCGTTAAGGTATTGAAAGAAGAAAGCAAAAAATAA

>cadA lysine decarboxylase [*Escherichia coli* str. K-12 substr. MG1655] H9R, K44R, T88S, E111G, M176V, Y230H

ATGAACGTTATTGCAATATTGAATCGCATGGGGGTTTATTTTAAAGAAGAAC
CCATCCGTGAACTTCATCGCGCGCTTGAACGTCTGAACTTCCAGATTGTTTA
CCCGAACGACCGTGACGACTTATTACGACTGATCGAAAACAATGCGCGTCT
GTGCGGCGTTATTTTTGACTGGGATAAATATAATCTCGAGCTGTGCGAAGAA
ATTAGCAAAATGAACGAGAACCTGCCGTTGTACGCGTTCGCTAATACGTATT
CCTTGCTCGATGTAAGCCTGAATGACCTGCGTTTACAGATTAGCTTCTTTGA
ATATGCGCTGGGTGCTGCTGGAGATATTGCTAATAAGATCAAGCAGACCACT
GACGAATATATCAACACTATTCTGCCTCCGCTGACTAAAGCACTGTTTAAAT
ATGTTCGTGAAGGTAAATATACTTTCTGTACTCCTGGTCACATGGGCGGTAC
TGCATTCCAGAAAAGCCCGGTAGGTAGCCTGTTCTATGATTTCTTTGGTCCG

67 AATACCGTAAAATCTGATATTTCCATTTCAGTATCTGAACTGGGTTCTCTGCT

68 GGATCACAGTGGTCCACACAAAGAAGCAGAACAGTATATCGCTCGCGTCTT

69 TAACGCAGACCGCAGCTACATGGTGACCAACGGTACTTCCACTGCGAACA

70 AAATTGTTGGTATGCATTCTGCTCCAGCAGGCAGCACCATTCTGATTGACCG

71 TAACTGCCACAAATCGCTGACCCACCTGATGATGATGAGCGATGTTACGCC

72 AATCTATTTCCGCCCGACCCGTAACGCTTACGGTATTCTTGGTGGTATCCCA

73 CAGAGTGAATTCCAGCACGCTACCATTGCTAAGCGCGTGAAAGAAACACC

74 AAACGCAACCTGGCCGGTACATGCTGTAATTACCAACTCTACCTATGATGGT

75 CTGCTGTACAACACCGACTTCATCAAGAAAACACTGGATGTGAAATCCATC

76 CACTTTGACTCCGCGTGGGTGCCTTACACCAACTTCTCACCGATTTACGAA

77 GGTAAATGCGGTATGAGCGGTGGCCGTGTAGAAGGGAAAGTGATTTACGA

78 AACCCAGTCCACTCACAAACTGCTGGCGGCGTTCTCTCAGGCTTCCATGAT

79 CCACGTTAAAGGTGACGTAAACGAAGAAACCTTTAACGAAGCCTACATGAT

80 GCACACCACCACTTCTCCGCACTACGGTATCGTGGCGTCCACTGAAACCGC

81 TGCGGCGATGATGAAAGGCAATGCAGGTAAGCGTCTGATCAACGGTTCTAT

82 TGAACGTGCGATCAAATTCCGTAAAGAGATCAAACGTCTGAGAACGGAAT

83 CTGATGGCTGGTTCTTTGATGTATGGCAGCCGGATCATATCGATACGACTGA

84 ATGCTGGCCGCTGCGTTCTGACAGCACCTGGCACGGCTTCAAAAACATCGA

85 TAACGAGCACATGTATCTTGACCCGATCAAAGTCACCCTGCTGACTCCGGG

86 GATGGAAAAAGACGGCACCATGAGCGACTTTGGTATTCCGGCCAGCATCGT

87 GGCGAAATACCTCGACGAACATGGCATCGTTGTTGAGAAAACCGGTCCGTA

88 TAACCTGCTGTTCCTGTTCAGCATCGGTATCGATAAGACCAAAGCACTGAG

CCTGCTGCGTGCTCTGACTGACTTTAAACGTGCGTTCGACCTGAACCTGCG
TGTGAAAAACATGCTGCCGTCTCTGTATCGTGAAGATCCTGAATTCTATGAA
AACATGCGTATTCAGGAACTGGCTCAGAATATCCACAAACTGATTGTTCAC
CACAATCTGCCGGATCTGATGTATCGCGCATTTGAAGTGCTGCCGACGATG
GTAATGACTCCGTATGCTGCATTCCAGAAAGAGCTGCACGGTATGACCGAA
GAAGTTTACCTCGACGAAATGGTAGGTCGTATTAACGCCAATATGATCCTTC
CGTACCCGCCGGGAGTTCCTCTGGTAATGCCGGGTGAAATGATCACCGAAG
AAAGCCGTCCGGTTCTGGAGTTCCTGCAGATGCTGTGTGAAATCGGCGCTC
ACTATCCGGGCTTTGAAACCGATATTCACGGTGCATACCGTCAGGCTGATGG
CCGCTATACCGTTAAGGTATTGAAAGAAGAAAGCAAAAAATAA

>ldcC lysine decarboxylase [*Escherichia coli* str. K-12 substr. MG1655]
ATGAACATCATTGCCATTATGGGACCGCATGGCGTCTTTTATAAAGATGAGC
CCATCAAAGAACTGGAGTCGGCGCTGGTGGCGCAAGGCTTTCAGATTATCT
GGCCACAAAACAGCGTTGATTTGCTGAAATTTATCGAGCATAACCCTCGAA
TTTGCGGCGTGATTTTTGACTGGGATGAGTACAGTCTCGATTTATGTAGCGA
TATCAATCAGCTTAATGAATATCTCCCGCTTTATGCCTTCATCAACACCCACT
CGACGATGGATGTCAGCGTGCAGGATATGCGGATGGCGCTCTGGTTTTTG
AATATGCGCTGGGGCAGGCGGAAGATATCGCCATTCGTATGCGTCAGTACA
CCGACGAATATCTTGATAACATTACACCGCCGTTCACGAAAGCCTTGTTTAC
CTACGTCAAAGAGCGGAAGTACACCTTTTGTACGCCGGGGCATATGGGCGG
CACCGCATATCAAAAAAGCCCGGTTGGCTGTCTGTTTTATGATTTTTTCGGC
GGGAATACTCTTAAGGCTGATGTCTCTATTTCGGTCACCGAGCTTGGTTCGT

112 TGCTCGACCACACCGGGCCACACCTGGAAGCGGAAGAGTACATCGCGCGG

113 ACTTTTGGCGCGGAACAGAGTTATATCGTTACCAACGGAACATCGACGTCG

114 AACAAAATTGTGGGTATGTACGCCGCGCCATCCGGCAGTACGCTGTTGATC

115 GACCGCAATTGTCATAAATCGCTGGCGCATCTGTTGATGATGAACGATGTAG

116 TGCCAGTCTGGCTGAAACCGACGCGTAATGCGTTGGGGATTCTTGGTGGGA

117 TCCCGCGCCGTGAATTTACTCGCGACAGCATCGAAGAGAAAGTCGCTGCTA

118 CCACGCAAGCACAATGGCCGGTTCATGCGGTGATCACCAACTCCACCTATG

119 ATGGCTTGCTCTACAACACCGACTGGATCAAACAGACGCTGGATGTCCCGT

120 CGATTCACTTCGATTCTGCCTGGGTGCCGTACACCCATTTTCATCCGATCTA

121 CCAGGGTAAAAGTGGTATGAGCGGCGAGCGTGTTGCGGGAAAAGTGATCT

122 TCGAAACGCAATCGACCCACAAAATGCTGGCGGCGTTATCGCAGGCTTCGC

123 TGATCCACATTAAAGGCGAGTATGACGAAGAGGCCTTTAACGAAGCCTTTA

124 TGATGCATACCACCACCTCGCCCAGTTATCCCATTGTTGCTTCGGTTGAGAC

125 GGCGGCGGCGATGCTGCGTGGTAATCCGGGCAAACGGCTGATTAACCGTTC

126 AGTAGAACGAGCTCTGCATTTTCGCAAAGAGGTCCAGCGGCTGCGGGAAG

127 AGTCTGACGGTTGGTTTTTCGATATCTGGCAACCGCCGCAGGTGGATGAAG

128 CCGAATGCTGGCCCGTTGCGCCTGGCGAACAGTGGCACGGCTTTAACGATG

129 CGGATGCCGATCATATGTTTCTCGATCCGGTTAAAGTCACTATTTTGACACC

130 GGGGATGGACGAGCAGGGCAATATGAGCGAGGAGGGGATCCCGGCGGCGC

131 TGGTAGCAAAATTCCTCGACGAACGTGGGATCGTAGTAGAGAAAACCGGC

132 CCTTATAACCTGCTGTTTCTCTTTAGTATTGGCATCGATAAAACCAAAGCAA

133 TGGGATTATTGCGTGGGTTGACGGAATTCAAACGCTCTTACGATCTCAACCT

GCGGATCAAAAATATGCTACCCGATCTCTATGCAGAAGATCCCGATTTCTAC
CGCAATATGCGTATTCAGGATCTGGCACAAGGGATCCATAAGCTGATTCGTA
AACACGATCTTCCCGGTTTGATGTTGCGGGCATTCGATACTTTGCCGGAGAT
GATCATGACGCCACATCAGGCATGGCAACGACAAATTAAAGGCGAAGTAG
AAACCATTGCGCTGGAACAACTGGTCGGTAGAGTATCGGCAAATATGATCC
TGCCTTATCCACCGGGCGTACCGCTGTTGATGCCTGGAGAAATGCTGACCA
AAGAGAGCCGCACAGTACTCGATTTTCTACTGATGCTTTGTTCCGTCGGGC
AACATTACCCCGGTTTTGAAACGGATATTCACGGCGCGAAACAGGACGAA
GACGGCGTTTACCGCGTACGAGTCCTAAAAATGGCGGGATAA

>KIVD decarboxylase mutant Lactococcus lactis subsp. lactis KF147 (strain: KF147, nat-host: mung bean, sub-species: lactis)

ATGTATACAGTAGGAGATTACCTATTAGACCGATTACACGAGTTAGGAATTG
AAGAAATTTTTGGAGTCCCTGGAGACTATAACTTACAATTTTTAGATCAAAT
TATTTCCCGCAAGGATATGAAATGGGTCGGAAATGCTAATGAATTAAATGCT
TCTTATATGGCTGATGGCTATGCTCGTACTAAAAAAGCTGCCGCATTTCTTAC
AACCTTTGGAGTAGGTGAATTGAGTGCAGTTAATGGATTAGCAGGAAGTTA
CGCCGAAAATTTACCAGTAGTAGAAATAGTGGGATCACCTACATCAAAAGT
CCAAAATGAAGGAAAATTTGTTCATCATACGCTGGCTGACGGTGATTTTAA
ACACTTTATGAAAATGCACGAACCTGTTACAGCAGCTCGAACTTTACTGAC
AGCAGAAAATGCAACCGTTGAAATTGACCGAGTACTTTCTGCACTACTAAA
AGAAAGAAAACCTGTCTATATCAACTTACCAGTTGATGTTGCTGCTGCAAA
AGCAGAGAAACCCTCACTCCCTTTGAAAAAAGAAAATCCAACTTCAAATA

CAAGTGACCAAGAGATTTTGAATAAAATTCAAGAAAGCTTGAAAAATGCCAAAAAACCAATCGTGATTACAGGACATGAAATAATTAGCTTTGGCTTAGAAAATACAGTCACTCAATTTATTTCAAAGACAAAACTCCCTATTACGACATTAAACTTTGGAAAAAGTTCAGTTGATGAAACTCTCCCTTCATTTTTAGGAATCTATAATGGTAAACTCTCAGAGCCTAATCTTAAAGAATTCGTGGAATCAGCCGACTTCATCCTGATGCTTGGAGTTAAACTCACAGACTCTTCAACAGGAGCATTTACCCATCATTTAAATGAAAATAAAATGATTTCACTGAACATAGACGAAGGAAAAATATTTAACGAAAGCATCCAAAATTTTGATTTTGAATCCCTCATCTCCTCTCTCTTAGACCTAAGCGGAATAGAATACAAAGGAAAATATATCGATAAAAAGCAAGAAGACTTTGTTCCATCAAATGCGCTTTTATCACAAGACCGCCTATGGCAAGCAGTTGAAAACCTAACTCAAAGCAATGAAACAATCGTTGCTGAACAAGGGACATCAGCCTTTGGCGCTTCATCAATTTTCTTAAAACCAAAGAGTCATTTTATTGGTCAACCCTTATGGGGATCAATTGGATATACATTCCCAGCAGCATTAGGAAGCCAAATTGCAGATAAAGAAAGCAGACACCTTTTATTTATTGGTGATGGTTCACTTCAACTTACAGTGCAAGAATTAGGATTAGCAATCAGAGAAAAAATTAATCCAATTTGCTTTATTATCAATAATGATGGTTATACAGCCGAAAGAGAAATTCATGGACCAAATCAAAGCTACAATGATATTCCAATGTGGAATTACTCAAAATTACCAGAATCATTTGGAGCAACAGAAGAACGAGTAGTCTCGAAAATCGTTAGAACTGAAAATGAATTTGTGTCTGTCATGAAAGAAGCTCAAGCAGATCCAAATAGAATGTACTGGATTGAGTTAGTTTTGGCAAAAGAAGATGCACCAAAAGTACTGAAAAAAGCGGGTAAACTATTTGCTGAACAAAATAAATCATAA
